# Early Loading of Single-Piece Implant for Partially Edentulous Posterior Arch: A Prospective One-Year Case Report

**DOI:** 10.1155/2013/854062

**Published:** 2013-04-08

**Authors:** Amol Beldar, Manohar L. Bhongade, Girish Byakod, Chandrashekar Buregoni

**Affiliations:** ^1^Department of Periodontics and Implant Dentistry, Mahatma Gandhi Vidyamandir's KBH Dental College, Panchavati, Nashik 422003, Maharshtra, India; ^2^Department of Periodontics and Implant Dentistry, Sharad Pawar Dental College and Hospital Of Deemed University, Sawangi (Meghe), Wardha, Maharashtra, India; ^3^Department of Periodontics and Implant Dentistry, Rangoonwala Dental College, Pune, Maharashtra, India

## Abstract

Implant therapy is now well established, and there is an increasing need for shorter rehabilitation time. Original prerequisites of osseointegration have been reassessed to satisfy continuously increasing patient's expectation of reduced treatment time, improved esthetics, and increased comfort. Shorter healing time may be appropriate in some circumstances, and examples of early loading have been reported in animal and human studies. However, to date there are insufficient data to determine a universally acceptable opinion on early loading of implants for single-tooth replacement. This case report involves early loading, combined with construction of a restoration, inserted directly after 6 weeks of implant surgery and followup of one year.

## 1. Introduction

Implants used for single-tooth replacement represent a more recent evolution of implant dentistry. As far greater number of patients are edentulous in a single-tooth gap or partial-arch space than completely edentulous. The opportunity to provide implant-supported tooth replacement for these patients significantly exceeds the opportunity for those who are completely edentulous [1]. The biomechanics of implants in these situations are significantly more different than in completely edentulous conditions, particularly in the context of early restoration of these implants. Abundant evidence clearly exists to support early loading of implants under full-arch clinical conditions. One of the unraveled parameters predicting osseointegration is micromotion at the implant-tissue interface not surpassing the threshold of 50–150 microns during the postimplantation healing phase. The most prerequisite for immediate loading and early loading is the achievement of high implant stability. Limiting implants micromotion below the threshold that could interfere with osseointegration, despite occlusal function, has been well documented and elucidated by means of authors [2].

Several long-term studies on single-tooth replacement have shown excellent results over a 5-year period [3]. To achieve successful bone-to-implant contact (osseointegration), oral implants placed according to a 2-stage surgical protocols have been advocated to remain unloaded for a healing period of 3–6 months [3]. A reanalysis of this original experimental design has questioned the necessity for a long implant healing period [3]. The current scientific literature supports the concept that implants can be loaded early or immediately. Recently, healing time of 6 weeks for implants placed in good quality bone has been recommended [4]. These recommendations resulted from a better understanding of the bone implant healing interface [5] and improved implant surface technology [6]. Studies regarding different types of prosthesis have shown that early loading of implants can provide treatment outcomes comparable to those achieved using standard healing periods before loading [7]. The early loading of implant supporting a full arch prosthesis in the edentulous mouth has also been studied. The studies regarding early loading of implant supported single tooth crowns in anterior arch are available in the literature but case reports and studies on early loading replacing single-tooth implant in posterior arch have not been reported. The purpose of the investigation was to know whether the early loading of single-tooth implant deleteriously affects the implant survival in the posterior arch.

## 2. Case Report

**Figure 1:**
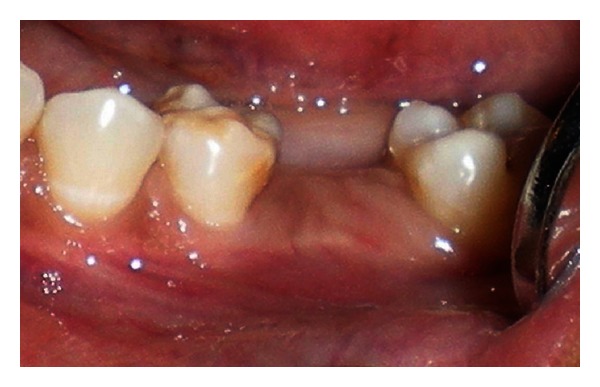
Preoperative view of the posterior left first molar, number 36.

**Figure 2:**
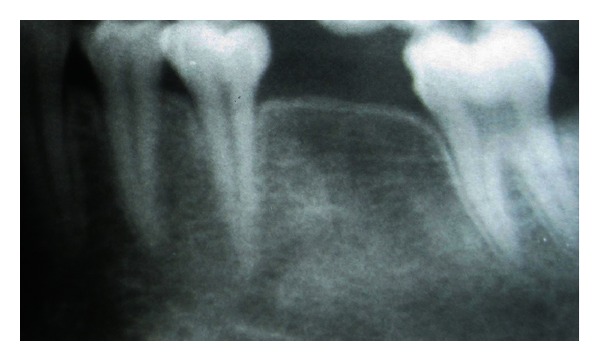
Preoperative radiograph of tooth, number 36, note the good height of the alveolar bone.

**Figure 3:**
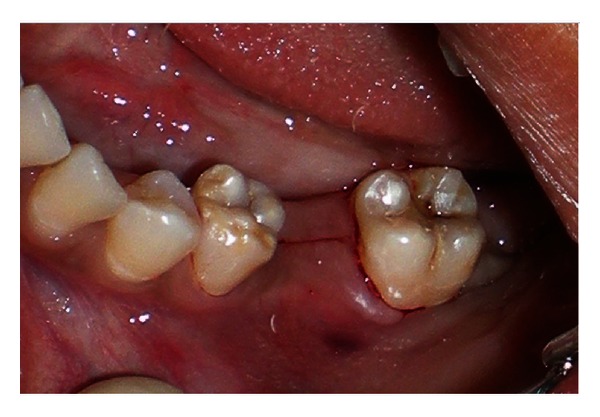
Crestal incision made along the crest of the ridge.

**Figure 4:**
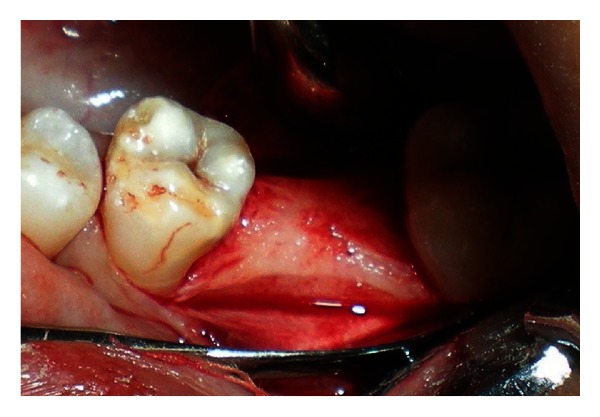
Broad buccolingual width of the residual ridge.

**Figure 5:**
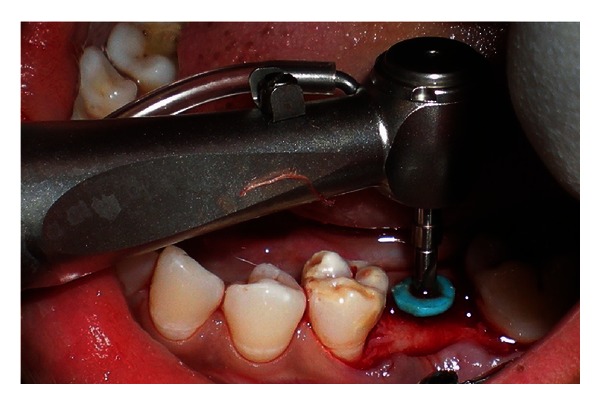
Pilot drill is used to increase the osteotomy site to confirm position and angulation.

**Figure 6:**
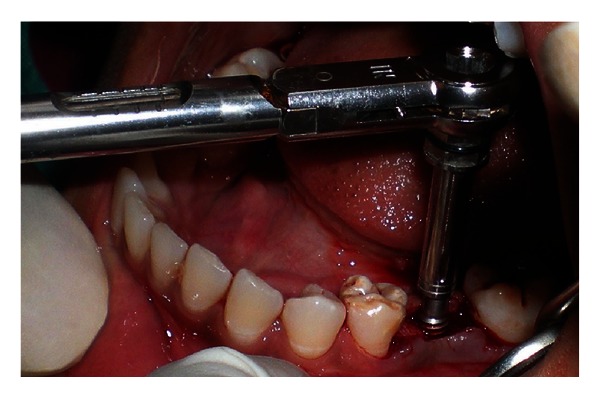
Wrench used for tightening implant.

**Figure 7:**
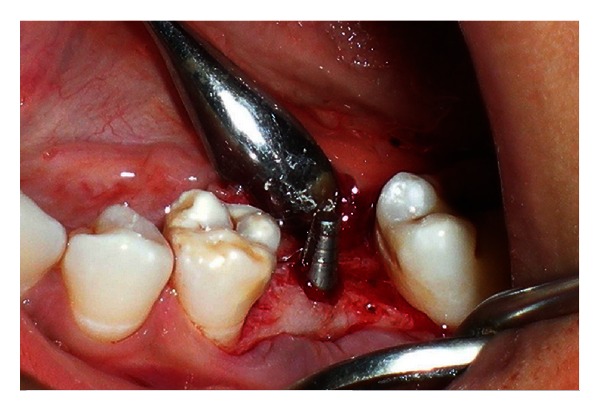
Implant placed into the osteotomy site.

**Figure 8:**
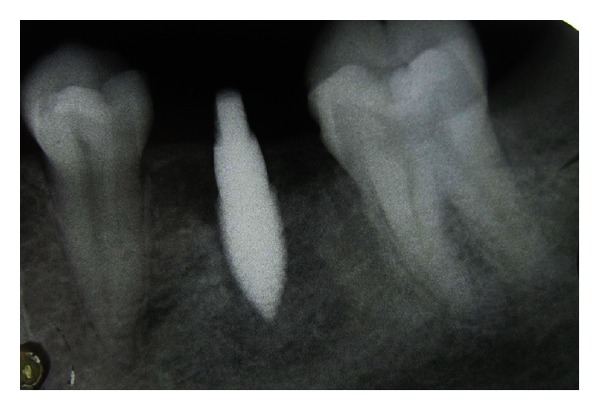
Radiographic view of the implant after surgery.

A 20-year-old female patient presented with a request to discuss the options for prosthetic replacement of mandibular left first molar which was extracted 6 months back. Clinical examination revealed a mesioangular impacted tooth third molar which was advised to be extracted. After two months, the patient reported back again for the prosthetic replacement of the molar (Figures 1 and 2). There was no significant past or present medical history and oral hygiene was acceptable. Prior to the surgical procedure, the patient was instructed to rinse with 0.2% chlorhexidine gluconate (Hexidine, ICPA Health Products Ltd., India) for one minute. The surgical protocols emphasized complete asepsis and infection control. The type of implant used was an acid-etched, tapered self threaded implant (HI-Tec) of diameter 4.5 × 13 mm. Briefly, after induction of local anesthesia, a crystal incision was made along the crest of the ridge using Bard-Parker number 12 surgical blade, bisecting the existing keratinized mucosa (Figure 3). A full thickness mucoperiosteal flap was raised buccally and lingually to the level of mucogingival junction, exposing the underlined ridge of the implant site, and a ridge alveoplasty was performed with the help of 0.2 mm round bur to achieve a flat bone surface of sufficient width (Figure 4). A surgical drill guide was used for the precise placement of the pilot drill. After pilot drill application, the implant site was prepared with the corresponding size of parallel drill (Figure 5). The implants were placed in the recipient site by means of an insertion device, and a torque driver set at 35 N cm which was used to evaluate primary stability of implant (Figure 6). It was the level of torque that the manufacturer recommended to be applied to the implant. To detect and prevent unfavorable rotation of implant, the implant was secured through a mounting sleeve that allowed manual detection of rotation. The appropriate position of the implant neck in both the vertical and horizontal dimensions was decisive. The implant neck was positioned at the crystal bone level or slightly submerged (Figure 7). Immediately after implant placement, the flap was replaced in its original position and sutured with nonresorbable suture (4-0) using a combination of inverted mattress and interrupted sutures. The inverted mattress sutures kept the bleeding edges of the flap close together, while the interrupted sutures sealed the edges. Intraoral periapical radiograph was taken immediately after surgery (Figure 8).

## 3. Prosthetic Reconstruction

**Figure 9:**
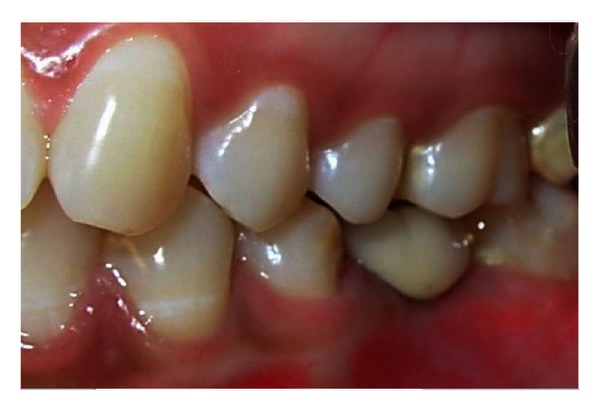
Clinical view of the final restoration at six weeks after surgery.

**Figure 10:**
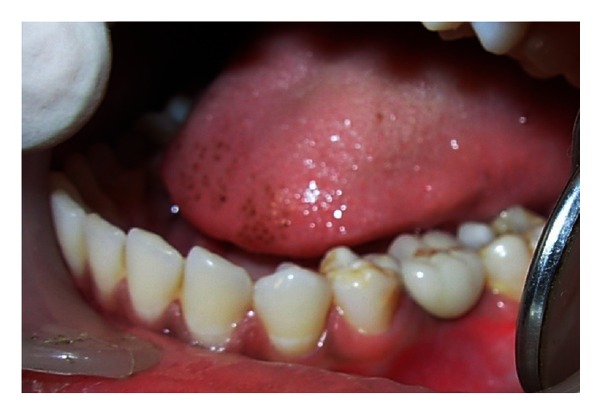
Clinical view of the final restoration at 12 months after surgery.

 After surgical intervention, the prefabricated temporary crown with acrylic resin was trimmed, polished, and cemented 24 hours after surgery. Compared to natural teeth, the temporary restoration was of a narrow occlusal surface without any contacts in functional occlusion. In maximum intercuspation, only point occlusal contacts were provided. The interproximal contacts were designed as broader contact areas to distribute the forces of mastication and to provide support. Depending on the gingival thickness, the crown margin was located from 0.5 to 1 mm below the gingiva. The temporary restoration was replaced with permanent restoration after six weeks (Figure 9). The definitive restoration was fabricated with a metal ceramic crown. The morphology of the occlusal surfaces of restoration was similar to that of the natural teeth with occlusal contact in maximum intercuspation and physiologic cusp inclination. The premature contacts during lateral and protrusive movement were avoided. The crown restoration was cemented and followup of one year of implant survival was assessed (Figure 10).

## 4. Discussion

 Most standard protocols in implant dentistry suggest a healing period of 3 months for mandible and 6 months for maxilla [8], but the time required for treatment, need for additional surgical procedures, and indefinite periods of temporization are obstacles that sometimes prevent the patients from implant treatments. To remove these obstacles, it would be beneficial to load implants within the few weeks after implant placement. Studies regarding different types of prostheses have shown that early loading of mandibular implants can provide treatment outcomes comparable to those achieved using standard healing periods before loading [9]. The long-term success of early loaded implant has been investigated in animal and human studies. Brånemark et al. [9] assessed peri-implant conditions of early loaded implants in a prospective split mouth controlled study and suggested that implant may very well be suitable for early loading at 6 weeks. Recently, Cochran et al. [10] in a prospective multicenter cohort study involving 133 patients with 383 implants found that implants could be successfully restored after 6 weeks of loading and yielded a success rate greater than 99%, two years after prosthetic restoration. The early loading of implants supporting a full arch prosthesis in edentulous mandible has also been studied. However, to the author's knowledge, studies regarding early loading of implants-supported single-tooth crowns in the mandible are not reported in the literature.

The basic concept behind the present case report was that early loading is not an absolute contraindication, but rather a relative one. Most available studies on the subject have offered solutions for full-arch reconstruction [11]. In such cases, the occlusal load is maximal and therefore requires maximal initial stability and support. For full-arch restorations, intra-arch stabilization is possible, and cross-arch stabilization is a recommended guideline to minimize micromovement, which can be a principal cause of early implant failure. For single-tooth restorations, the adjacent teeth can withstand a major part of the occlusal forces. In the present case report, acrylic resin temporary crowns were used to prevent transmission of some of the load directly to the implant and thicker acrylic resin occlusal width, but no more than 2 to 3 mm was used to further diminish the occlusal forces. Several factors influence stability: the potential bone-implant surface area (as dictated by length, width, and screw type versus cylinder, and microtexture); bone quality; initial bone-implant contact.

Early publications on immediate restoration of single, unsplinted implants in the esthetic zone were presented as case reports and series. Kupeyan and May [12] reported on series of 10 and 14 immediately restored implants, respectively, in the maxillary anterior region. Kupeyan and May [12] performed their study in healed ridges with machined titanium Brånemark System implants (Nobel Biocare), and all implants clinically integrated and remained stable for the observation periods of 6 months to 3 years. Additional case reports of small series of patients by Andersen and coworkers [13] and Cannizzaro and Leone (2003) [14] confirmed the observations of 100% survival of single-tooth replacement in the maxillary anterior region. All authors advocated maximization of implant stability by using long implants and eliminating occlusal contact in centric and excursive movements.

In the present case report, sandblasted acid etched (SLA) implant was used for early loading procedure. The advantages of SLA include faster osseointegration, proportionally greater bone-implant contact, and greater reverse-torque resistance compared to noncoated implants. It was also demonstrated that when single-tooth implant is placed in partially edentulous arch and loaded early (6 weeks), did not appear to jeopardize the osseointegration healing process in the posterior mandible. The primary and the secondary stability were also achieved. The primary implant stability at placement is the mechanical phenomenon related to the quality and quantity of the bone at the recipient site, the type and design of the implant used and the surgical technique used. The secondary implant stability increased in stability attributable to the bone formation and remodeling at the implant tissue interface in the surrounding bone. Piattelli et al. [15] compared the histology of nonsubmerged unloaded and early loaded titanium screw implants in monkeys. They found a tight contact of new bone to implant surfaces in all the samples examined. However, around the necks of early loaded implants, they observed lamellar cortical bone that was thicker than that in the unloaded implant. Engquist et al. [16] found less marginal bone loss in early loaded implants as compared to conventional implants. If implants are placed in soft bone, initial stabilization can be compromised leading to micromotion and failure. Immediate/early stabilization and splinting of implants help to reduce excessive micromotion of implants. 

Excellent short-term data have been presented for early loaded implants in partially edentulous jaws. However, it must be remembered that most of the papers reviewed are produced by practitioners who are highly trained in dental implant placement. Few long-term multicenter studies are available. Most studies on patient benefits are also needed. Besides shorter treatment time for the doctor/patients with early loaded implants, there are psychological factors for the patients that warrant more attention. Based on this case report, it is suggested that the immediate loading/early method should be limited to healed sites. Further clinical and histologic studies are necessary to promote routine clinical application of this technique.

## 5. Conclusions

Implants with high initial primary stability seem to function well under the influence of immediate and early loading. Available bone quality needs to be evaluated to ensure the proper implant diameter. By using surgical methods capable of enhancing primary implant stability, the placement of early loaded implants in less dense bone can result in a successful outcome. A successful integration of early loaded implants may require a final torque exceeding 35 N cm and an implant stability quotient value above 60. No difference in bone remodeling seems to exist between early loaded and two-stage implants. Within the limits of the present investigation, early loading of single-tooth implants placed in healed sites was a possible treatment alternative. Obviously, long-term data are needed to fully evaluate the benefits and risks of early loaded implants.
